# Bioactive potential of *Streptomyces* against fish and shellfish pathogens

**Published:** 2010-09

**Authors:** D Selvakumar, K Arun, S Suguna, D Kumar, K Dhevendaran

**Affiliations:** 1 Department of Aquatic Biology and Fisheries, University of Kerala, Kariavattom campus, Trivandrum, 695 581, Kerala, India; 2 Department of Biotechnology, AVVM Sri Pushpam College, Poondi, Thanjavur, 613 503, Tamil Nadu, India

**Keywords:** Marine Sponges, Streptomyces, Bioactive compounds, Fish and shellfish pathogens

## Abstract

**Background and Objectives:**

In the present study, isolation of Streptomyces associated with marine sponges and its bioactive potential against fish and shellfish pathogens were assessed. The *Streptomyces* sp. were isolated from the marine sponges namely *Callyspongia diffusa*, *Mycale mytilorum*, *Tedania anhelans* and *Dysidea fragilis* collected from Vizhinjam port, situated in the South-West coast of India.

**Materials and Methods:**

The Streptomyces associated with marine sponges were isolated using specific ISP media. The isolates of *Streptomyces* were characterized for their colony characteristics, morphological properties, physiological and biochemical properties and were tentatively identified. The strains were cultivated on a lab scale level as shake-flask cultures and the crude extracts of the bioactive compounds obtained with ethyl acetate were screened biologically and chemically. By biological screening, the extracts were analyzed for their activity against fish and shellfish pathogens namely *Aeromonas hydrophila*, *Serratia* sp. and *Vibrio* spp, using the disk and agar-well diffusion bioassay method, while by chemical screening the crude culture extracts were analyzed by TLC and UV–Vis spectrophotometer.

**Results:**

Ninety-four isolates were found to be associated with marine sponges, among them only seven strains showed antagonism against fish and shellfish pathogens. Analysis of morphological, physiological and biochemical characteristics suggested that these strains belonged to the genus Streptomyces. The initial screening of the isolates by spot inoculation method exhibited antibacterial activity against *Aeromonas hydrophila*. *In-vitro* screening of the submerge culture extracts showed positive inhibition against the fish and shellfish pathogens namely *Aeromonas hydrophila*, *Serratia* sp. and *Vibrio* spp. The screening of bioactive compounds confirmed the production of polyene substances by UV spectrum, which resulted in absorbance peaks ranging from 225 to 245 nm and TLC analysis yielded R_f_ values ranging from 0.40 to 0.78.

**Conclusion:**

The results suggest that the seven Streptomyces strains isolated from marine sponges produce potential antibacterial compounds against fish and shellfish pathogens.

## INTRODUCTION

Marine microorganisms are of considerable interest as a new and promising source of biologically active compounds (1). They produce a variety of metabolites, some of which can be used for drug development (2). Marine Actinomycetes are a very important group of bacteria that have considerable value as prolific producers of antibiotics and other therapeutic compounds. Actinomycetes produce over half of the bioactive compounds in nature (3). The isolation of actinomycetes from marine environments has been a fruitful area of research in the past decade. However, little is known about the diversity of actinomycetes from marine samples compared to the diverse range of actinomycetes isolated from terrestrial environments (4). Increasing numbers of both culture-based studies and culture-independent molecular studies showed that many actinomycetes exist in marine environments such as sediments, seawater, and marine invertebrates (5, 6).

Many marine actinomycetes taxon in ocean sediments were found to have widespread, persistent populations in ocean systems and some taxa have no counterparts in terrestrial environments. It is becoming evident that marine habitats are an abundant source of actinomycetes for discovering natural products. Many promising bioactive compounds including antimicrobial, antitumour, immunosuppressive agents and enzymes are being discovered from marine actinomycetes (7). The antibiotics produced are entirely new and unique when compared to those from the terrestrial ones. Of the marine inhabitants investigated, marine invertebrates, particularly sponges, are of great interest for discovering novel actinomycetes. As sessile filter-feeding animals, sponges are the largest sources of marine bioactive metabolites, accounting for up to 40% of all known natural marine products (8). However, few studies have investigated the diversity, distribution and ecology of actinomycetes from marine sponges, although there are several reports that they are abundant (9). There are several reports on the bioactivity of actinomycetes namely Streptomyces from marine sediments against human and fish pathogens (10, 11). But only few studies have focused on sponges associated Streptomyces against human pathogens (12). In the present investigation, the antagonistic potential of sponges associated with actinomycetes, namely Streptomyces, against fish and shellfish pathogens were assessed.

## MATERIALS AND METHODS

**Microorganism and maintenance of culture.** The microorganisms used in the study were *Streptomyces* strains isolated from the marine sponges namely *Callyspongia diffusa, Mycale mytilorum, Tedania anhelans,* and *Dysidea fragilis*. Identification of the marine sponges and isolation of *Streptomyces* strains were mentioned in an earlier report (12). The isolated strains were maintained as glycerol asparagine agar slant cultures at 28 ± 2°C. The inocula used in all the experiments were seven day-old cultures, unless otherwise stated.

**Characterisation of the Streptomyces isolates.** The strains were preliminarily characterised by the method of International Streptomyces Project (ISP) (13). The microorganism were characterised by acid-fast staining and Gram's staining techniques. The isolates were also studied by employing various parameters which are detailed below.

**Pigmentation of mycelia and spore morphology.** The cultures were grown on a Petri dish containing casein-starch-peptone-yeast extract (CSPY) agar medium with a cover slip inserted at an angle of 45°. The cover slip was removed after 7 days of incubation, air dried and observed under scanning electron microscope (14).

**Melanoid production.** The cultures were streaked onto peptone-yeast extract-iron agar slants and incubated at 28°C for 48 h.

**Utilization of carbon sources.** Various carbon sources namely glucose, xylose, arabinose, rhamnose, fructose, galactose, raffinose, mannitol, inositol, and sucrose were mixed at a concentration of 1% each to 10 ml of basal mineral salt medium and incubated at 28°C for 7 days.

**Influence of amino acids.** Various amino acids namely glycine, cystine, alanine, tryptophan, and valine were mixed at a concentration of 0.1% each to 5 ml of basal mineral salt medium and incubated at 28°C for 7 days. The biomass thus obtained was separated from the broth, dried and weighed. The weight of the biomass was expressed in grams.

**Sodium chloride tolerance.** Sodium chloride at varying concentrations (1, 1.5, 2, 2.5, 3, 4, 5, 6, 7, 8, 9 and 10%) were added to 5 ml of the basal medium and incubated at 28°C for 7 days. The biomass thus obtained was separated from the broth, dried and weighed. The weight of the biomass was expressed in grams.

Physiological and biochemical characteristics were studied according to the procedures described by Buchanan and Gibbons (15).

**Anti microbial assay: Spot inoculation method.** Preliminary screenings of bioactivity were done by the method described (16). The strains were spot inoculated in glycerol asparagine medium for seven days. After seven days, one ml of chloroform was added and made to stand for 40 minutes to arrest the growth of inoculated colonies and then they were overlaid with 5ml of sloppy agar (0.6%) layer previously seeded with anyone of the test organism. Here, the selected test organism was *Aeromonas hydrophila.* It were incubated for 24 hours at 37°C and the diameter of the incubation zone was recorded in millimetres.

***In-vitro* screening of isolates for antimicrobial activity (Disc method).** The isolates were inoculated as submerged culture in 500 ml Erlenmeyer flasks containing 100 ml of the liquid medium (0.8 g NaCl, 1 g NH_4_Cl, 0.1 g KCl, 0.1 g KH_2_PO_4_, 0.2 g MgSO_4_.7H_2_O, 0.04 g CaCl_2_.2H_2_O, 2 g glucose, 3 g yeast extract in one litre of distilled water, pH 7.3). These cultures were grown in a rotary shaker at 200 rpm, 28°C for 120 hours under the standard conditions of aeration and agitation. The resultant cultures were centrifuged for 15 minutes. The culture filtrates were solvent extracted with ethyl acetate (1:1) in the separating funnel and shaken vigorously for 20 minutes. The upper organic layers were collected and evaporated to dryness in a vacuum evaporator at 40°C. A crude gummy extract was obtained. The crude extracts were suspended in ethyl acetate at a concentration of 1 mg per ml for antimicrobial studies. Sterile filter paper discs, 6 mm in diameter (Hi Media, India), were impregnated with 50 µl (50 µg crude extract) suspension, dried and placed onto the plates previously seeded with fish and shellfish pathogens namely *Aeromonas hydrophila*, *Serratia* sp. and *Vibrio* spp. The selective medium Thiosulphate–citrate–bile salts–sucrose (TCBS) medium was used for cultivation of *Vibrio* spp. (*V*. *alginolyticus*, *V. harveyi* and *V. parahaemolyticus*). Tryptone soya agar medium was used for culturing *Aeromonas hydrophila* and nutrient agar medium was used for *Serratia* sp. The plates were incubated for 24 hrs at 37°C for *Aeromonas hydrophila*, *Serratia* sp. and 24 hrs at 28°C for *Vibrio* spp. and the diameter of inhibition zones were measured (17).

**Screening of polyene substances from the submerged culture extracts.** Polyene compounds were recovered from the culture filtrates by solvent extraction with ethyl acetate in 1:1 (v/v) ratio and shaking for 1 hour (18). The ethyl acetate phase was separated and evaporated to dryness in water bath at 80 – 90°C and the residue was weighed and redissolved with little volume of ethyl acetate. The absorption spectrum was determined by UV and Visible light (200 – 600 nm) by using UV/VIS spectrophotometer 2101 (Systronics).

Thin layer chromatographic analysis of antibacterial compounds. The extracts were spotted on the baseline of the silica gel plates (stationary phase) at 1 cm and then allowed to dry at room temperature (19). The plates were placed in TLC chamber pre-saturated with the mobile phase butanol: acetic acid: water (4:1:2). The chromatogram was developed and was visualized under UV light and the spots were marked. The R_f_ values for each spot was measured.

## RESULTS

In the present investigation, bioactive potential of marine sponges associated *Streptomyces* against fish and shellfish pathogens were assessed. The isolation of *Streptomyces* was carried out using the selective media, glycerol asparagine agar (ISP-5).

Nearly 94 cultures of *Streptomyces* were isolated from the four marine sponges. Among the ninety-four cultures, only seven strains exhibited positive antagonism against fish and shellfish pathogens. The preliminary characterizations were carried out by the methods recommended by International Streptomyces Project (ISP). The isolated strains were characterized by morphological, physiological and biochemical properties. The colonies were slow growing, chalky, folded and aerobic. The strains were acid-fast negative and found to be Gram-positive. The aerial mycelial colo r pattern of the strains were found to be white (AQBCD11, AQBCD24, AQBMM35, AQBTA66), grey (AQBCD03), grey white (AQBDF81), yellow (AQBMM49) and the vegetative mycelial colors were white (AQBCD11, AQBMM49), yellow (AQBCD03, AQBDF81), pale yellow (AQBMM35), orange (AQBCD24), and red (AQBTA66). All the strains do not produce melanoid pigments. Studies on spore morphology revealed that the strains bore rectus flexibiles hyphae and smooth sporephores.

The results of physiological and biochemical characteristics of the strains are displayed in Table 1. All the strains were able to grow in 22–45°C and pH 4–10. The strains were able to liquefy gelatin except AQBMM49 and AQBTA66. Solidification of milk and peptone cannot be done by strain AQBCD24. Strains AQBMM35 and AQBMM49 were able to hydrolyze starch and cellulose but were unable to produce hydrogen sulphide. Degradation of urea was effectively done by AQBCD11 and AQBMM35. Positive utilization of citrate was confirmed in the strains AQBCD03, AQBCD11, AQBMM35, AQBMM49, AQBTA66, other than AQBCD24, AQBDF81. Indole productions were not seen in strains AQBMM35 and AQBMM49. Lastly, the catalyse activity was effectively seen in all strains other than AQBCD11 and AQBMM35.

**Table 1:** Biochemical properties and carbon utilization of Streptomyces strains (+ Positive results,-Negative results, ± Doubtful results)

Biochemical parameters	AQBCD03	AQBCD11	AQBCD24	AQBMM35	AQBMM49	AQBTA66	AQBDF81
Starch hydrolysis	−	−	−	+	+	−	−
Production of H_2_S	+	+	+	−	−	+	+
Degradation of cellulose	−	−	−	+	+	−	−
Liquefaction of gelatin	+	+	+	+	−	−	+
Coagulation of milk	+	+	−	+	+	+	+
Peptonization of milk	+	+	−	+	+	+	+
Degradation of urea	−	+	−	+	−	−	−
Citrate utilization	+	+	−	+	+	+	−
Indole production	+	+	+	−	−	+	+
Catalase	+	−	+	−	+	+	+
							

**Carbon source utilization**							

No carbon source (negative control)	−	−	−	−	−	−	−
Xylose	+	+	+	+	+	+	+
Arabinose	+	+	−	+	+	+	−
Rhamnose	+	−	+	−	−	−	−
Fructose	+	+	+	−	−	+	+
Galactose	−	−	+	+	+	+	+
Raffinose	−	+	−	+	−	−	−
Mannitol	+	−	−	−	+	+	+
Inositol	−	−	−	−	−	−	−
Sucrose	−	−	±	±	−	±	±
Glucose (positive control)	+	+	+	+	+	+	+

**Fig. 1:**
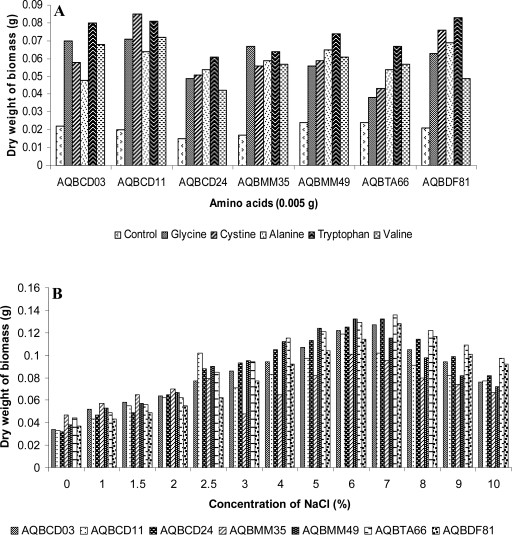
Influence of amino acids (A) and sodium chloride concentrations (B) on the growth of the seven strains.

The nutritional characteristics of the strains were studied using criteria like carbon utilization, amino acids influence and sodium chloride tolerance. The utilization of carbon sources is displayed in Table 1. The strains grew well in media containing glucose and xylose but the strains did not assimilate to inositol. On medium with sucrose, the growth of the strains were weak or absent. Amino acids, glycine and tryptophan strains except the strain AQBCD11 which showed better growth in media containing cystine (Fig. 1A). At the sodium chloride concentration of 6 and 7%, the strains showed profuse growth. The strains AQBCD11, AQBMM35 and AQBMM49 exhibited maximal biomass at concentration of 6%, whereas the rest of the strains showed maximal biomass at 7% (Fig. 1B).

**Fig. 2:**
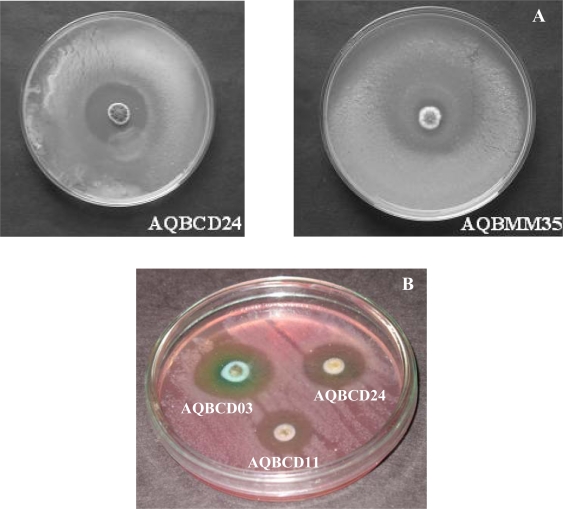
Inhibition zone of the strains against *Aeromonas hydrophila* (A) by Spot inoculation method and In vitro screening against *Vibrio harveyi* (B) by Disc method.

Preliminary identification of antagonism against pathogen *Aeromonas hydrophila* was done by the spot inoculation method, in which the strains showed more than 10 mm of inhibition zone (Fig. 2A). Later, *in-vitro* screening of the culture extracts was carried out by disc method which resulted in more than 10 to 30 mm diameters of inhibition zone (Table 2). Above 30 mm inhibition zone was recorded against *Aeromonas hydrophila, Vibrio parahaemolyticus* and *Vibrio harveyi*. The strains AQBCD03, AQBCD24, AQBMM35, and AQBDF81 exhibited less activity at 10 mm against *V. parahaemolyticus*, *V. alginolyticus*, and *V. harveyi*. Some strains showed inhibition against *V. harveyi* which are displayed (Fig. 2B).

**Table 2:** Antagonistic activity of Streptomyces strains against fish pathogens (Inhibition zone: +,<10 mm; ++, 10–20 mm; +++, 21–30 mm; ++++,>30 mm;-No Inhibition zone).

Strains	Disc method					Spot inoculation method

	*Aeromonas hydrophila*	*Serratia sp.*	*Vibrio harveyi*	*Vibrio parahaemolyticus*	*Vibrio alginolyticus*	
**AQBCD03**	++++	++	++++	+	+++	++++
**AQBCD11**	++	++	+++	++	+++	+++
**AQBCD24**	++	++	+++	++++	+	++++
**AQBMM35**	++++	++++	++	++	+	++++
**AQBMM49**	+++	+++	++	+	++	+++
**AQBTA66**	++	+++	++	+++	++	+++
**AQBDF81**	+++	+	++	++	++	++

The screenings of bioactive compounds were carried out from the submerged culture extracts of the seven strains by UV spectral and thin layer chromatographic analysis. The UV spectral analysis resulted in maximum absorbance peaks ranging from 225 to 262 nm and thin layer chromatographic analyses showed the R_f_ values were in the range of 0.40–0.78 (Table 3).

**Table 3:** UV absorption and R_f_ values of the culture extracts of Streptomyces isolates.

Strain	Maximum (nm)/ Absorbance values	Shoulder (nm)/ Absorbance values	R_f_ values
**AQBCD03**	237.8–2218	275.6–1.498	0.40, 0.46,0.78
		326–1.102	
**AQBCD11**	245–2.400	329.6–0.876	0.61, 0.77
**AQBCD24**	225.2–0.350	329.6–0.019	0.50, 0.54
**AQBMM35**	228.8–1.064	329.6–0.307	0.46, 0.61
**AQBMM49**	241.4–2.148	278.6–1.221	0.51, 0.63, 0.70
		332.2–0.264	
**AQBTA66**	245–2.290	331.4–0.437	0.51, 0.77, 0.78
		365.6–0.772	
**AQBDF81**	236–1.431	282.8–0.240	0.46, 0.51

## DISCUSSION

In this study, nearly 94 strains were found to be associated with four sponges and screened for bioactivity against fish and shellfish pathogens. But only seven cultures showed effective bioactivity against fish and shellfish pathogens. Preliminarily, the seven strains were characterized for morphological appearance of the aerial mycelia, vegetative mycelium, sporophore, and spores. The mycelial colors of the seven isolates were totally different and they were classified accordingly. This mycelial color difference may be due to the synthesis of various secondary metabolites (20). These secondary metabolites are compounds consisting of amino acids, sugars, fatty acids, terpenes, etc. The strains showed typical morphology of *Streptomycetes* when analyzing the shape and spore chains under scanning electron microscope when compared with the earlier reports (14). The strains were further characterized for physiological and biochemical properties, nutritional uptake and all the isolates fitted the *Streptomyces* genus as reported by several investigators (13). The identification of the *Streptomyces* is a very complex process. The *Streptomyces* classification system was mainly dependent on characteristics like the form of spores, melanoma and use of carbon (15).

Earlier reports on the ninety-four cultures associated with four marine sponges produced antibacterial and antifungal activity against human pathogens (12). In the present study, bioactivity of the strains against fish and shellfish were assessed. Initial screening revealed effective antagonism against fish pathogen namely *Aeromonas hydrophila* by spot inoculation method and further in vitro screening of the strains showed bioactivity against fish and shellfish pathogens by disc method. The fish and shellfish pathogens (*Aeromonas hydrophila, Serratia* sp., *Vibrio* spp.) used in the study have caused serious trouble for aqua-culturists for the past several decades. The fish pathogen *Aeromonas hydrophila* causes diseases like motile aeromonas septicemia, hemorrhagic septicemia and ulcer disease or red-sore disease (21). There are several strains of *Serratia* causing disease in fish and even causing food poisoning to humans upon consuming sea food which has previously been affected (22). The major disease to shrimp aquaculture includes vibriosis caused by Vibrio species namely *Vibrio alginolyticus*, *V. harveyi* and *V. parahaemolyticus* (23, 24). There are some reports on actinomycetes strains from marine sediments against shrimp pathogens like *Vibrio* spp (25).

The screening of bioactive compounds through UV spectral data and TLC R_f_ values confirmed the production of polyene substances. UV absorbance spectrum and TLC data were clearly explained in the previous report and showed a differential pattern for each isolate (12). This might indicate that these isolates produce different bioactive compounds involved in the inhibition of particular fish and shellfish pathogens. The spectral data and TLC R_f_ values were consistent with results obtained earlier (18, 26). It is quite obvious that the *Streptomyces* are inherent in marine sponges synthesizing commercially valuable bioactive compounds. Interestingly, in at least some cases, the antibacterial compounds appear to be produced in associated microorganisms rather than by the sponge (27).

It is concluded that marine sponge-associated *Streptomyces* strains represent a promising source of antibacterial agents against fish and shellfish pathogens. The result of active extracts from the antibacterial studies, and spectral and thin layer chromatographic analysis revealed that these strains were effective producers of bioactive metabolites against specific target pathogens. There are very limited reports on use of marine actinomycetes as antibacterial agents against fish pathogens. The discovery of new classes of antibiotics is highly necessary due to the increased incidence of resistant pathogens to drugs that are currently in use. Antibiotics developed from marine microbes are particularly important because they have high potency when compared with terrestrial counterparts.
